# The spatial distribution of pet dogs and pet cats on the island of Ireland

**DOI:** 10.1186/1746-6148-7-28

**Published:** 2011-06-10

**Authors:** Martin J Downes, Tracy A Clegg, Daniel M Collins, Guy McGrath, Simon J More

**Affiliations:** 1 Centre for Veterinary Epidemiology and Risk Analysis, Veterinary Sciences Centre, UCD School of Agriculture, Food Science and Veterinary Medicine, University College Dublin, Belfield, Dublin 4, Ireland

## Abstract

**Background:**

There is considerable international research regarding the link between human demographics and pet ownership. In several international studies, pet ownership was associated with household demographics including: the presence of children in the household, urban/rural location, level of education and age/family structure. What is lacking across all these studies, however, is an understanding of how these pets are spatially distributed throughout the regions under study. This paper describes the spatial distribution of pet dog and pet cat owning households on the island of Ireland.

**Results:**

In 2006, there were an estimated 640,620 pet dog owning households and 215,542 pet cat owning households in Ireland. These estimates are derived from logistic regression modelling, based on household composition to determine pet dog ownership and the type of house to determine pet cat ownership. Results are presented using chloropleth maps. There is a higher density of pet dog owning households in the east of Ireland and in the cities than the west of Ireland and rural areas. However, in urban districts there are a lower proportion of households owning pet dogs than in rural districts. There are more households with cats in the urban areas, but the proportion of households with cats is greater in rural areas.

**Conclusions:**

The difference in spatial distribution of dog ownership is a reflection of a generally higher density of households in the east of Ireland and in major cities. The higher proportion of ownership in the west is understandable given the higher proportion of farmers and rural dwellings in this area. Spatial representation allows us to visualise the impact of human household distribution on the density of both pet dogs and pet cats on the island of Ireland. This information can be used when analysing risk of disease spread, for market research and for instigating veterinary care.

## Background

There is considerable international research about the link between human demographics and pet ownership, and published data are available from several countries including the United Kingdom (UK) [1-3], the USA [4], Italy [5] and Brazil [6]. Demographic studies have been used to predict the usage of veterinary services [7-9] and future pet population trends [10], and to aid in managing pets (dogs in particular) for zoonotic disease control, especially rabies [8,11]. Pet ownership has been linked to several factors relating to household demographics, including the presence of children in the household [1,7,10], urban/rural location [1,7,12], level of education [13] and age/family structure [1,3,13].

There is very little published information about the demography of domestic pets on the island of Ireland, which incorporates both the Republic of Ireland (ROI) and Northern Ireland (NI). The number and location of pets (especially dogs) is currently of interest, particularly within the government and veterinary organisations in Ireland, with increasing awareness of zoonotic diseases [14-17], human dog interactions [18] and the introduction of a pet passport scheme [19]; negating the necessity for a 6 month quarantine period for transport of dogs into Ireland from certain designated countries. Market research has been used by the pet food industry to provide a descriptive view of pet ownership in Ireland, but has not sought associations with human demographics [20]. In an earlier study on the island of Ireland, we identified links between dog ownership and a number of demographic factors, including urban/rural location, house type, household social class, household composition and the presence of school children in the house [21]. This earlier study also examined the demographic links with pet cat ownership, which included the type of house structure, the gender and the age of the participant. As yet, however, there is little understanding of the spatial distribution of pets throughout the regions where these data were collected. With all this in mind, the current paper describes the spatial distribution of pet dog and pet cat owning households on the island of Ireland (for brevity, subsequently referred to as 'Ireland').

## Results

There were an estimated 2,142,121 human households in Ireland in 2006.

Pet dog ownership was significantly associated with household composition, being significantly higher in lone adult households with children (odds ratio [OR]: 3.26; 95% CI: 1.86, 5.73) compared with single occupancy households (Table 1). Pet cat ownership was significantly associated with house type, being lower in people who lived in an apartment or flat (OR: 0.11; 95% CI: 0.01, 0.77) compared with people who lived in a house (Table 2). The estimated number of pet dog and pet cat owning households in Ireland in 2006 was 640,620 and 215,542, respectively.

**Table 1:** The final logistic model of pet dog ownership by household composition

Variable name	Variable category	OR	95% CI	PP
Household composition	Single person households	1.00	-	0.2055
	Two adults without children	1.43	0.92, 2.22	0.2696
	Other households without children	1.47	0.77, 2.79	0.2753
	Lone adult households with children	3.26	1.86, 5.73	0.4578
	Other households with children	2.98	2.01, 4.42	0.4352

The final logistic model of pet dog ownership, including odds ratios (OR) and predicted probabilities of pet dog ownership (PP), by household composition. The data were collected using a telephone survey of 1,250 households on the island of Ireland in November 2007.

**Table 2:** The final logistic model of pet cat ownership by house type

Variable name	Variable category	OR	95% CI	PP
House type	House	1.00	-	0.1116
	Apartment/Flat	0.11	0.01, 0.77	0.0132
	Other	0.31	0.04, 2.28	0.0371

The final logistic model of pet cat ownership, including odds ratios (OR) and predicted probabilities of pet cat ownership (PP), by house type. The data were collected using a telephone survey of 1,250 households on the island of Ireland in November 2007.

**Figure 1:**
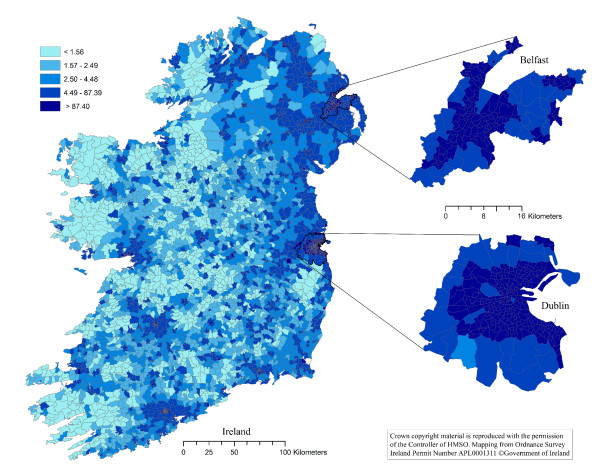
**The distribution pet dog owning households in Ireland**. The map shows the number of pet dog owning households per square kilometre in each electoral unit on the island of Ireland. Based on data collected using a telephone survey of 1,250 households in November 2007 and on data from the Central Statistics Office in the Republic of Ireland and the Northern Ireland Statistics and Research Agency.

**Figure 2:**
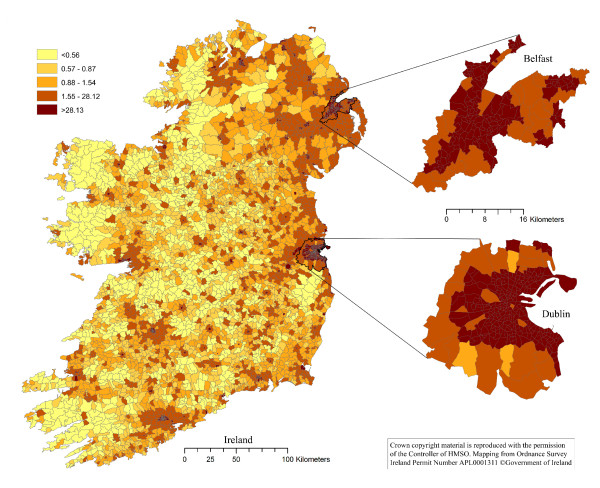
**The distribution pet cat owning households in Ireland**. The map shows the number of pet cat owning households per square kilometre in each electoral unit on the island of Ireland. Based on data collected using a telephone survey of 1,250 households in November 2007 and on data from the Central Statistics Office in the Republic of Ireland and the Northern Ireland Statistics and Research Agency.

**Figure 3:**
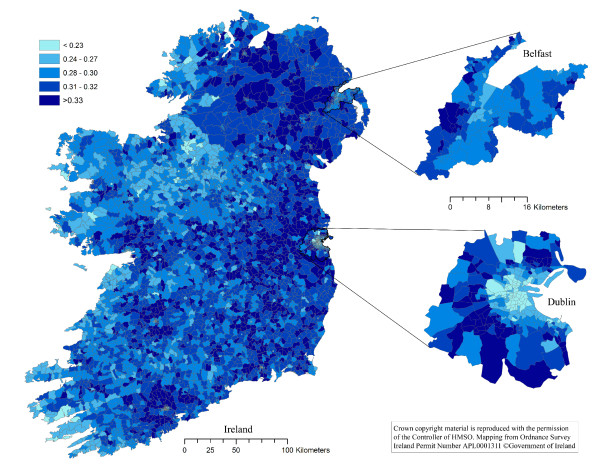
**The proportion of households in Ireland owning a pet dog**. The proportion of households on the island of Ireland owning a pet dog, by electoral unit. Based on data collected using a telephone survey of 1,250 households in November 2007 and on data from the Central Statistics Office in the Republic of Ireland and the Northern Ireland Statistics and Research Agency.

**Figure 4:**
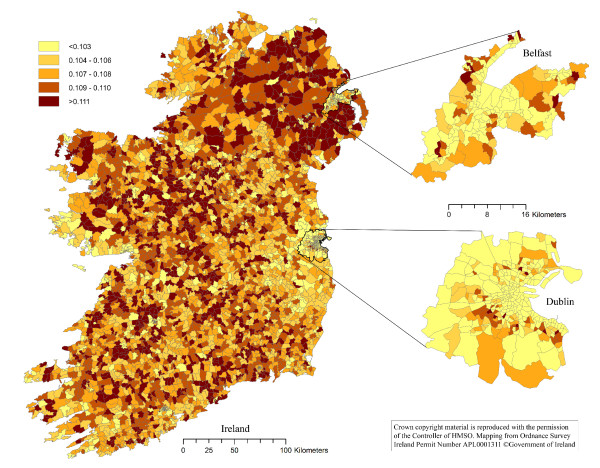
**The proportion of households in Ireland owning a pet cat**. The proportion of households on the island of Ireland owning a pet cat, by electoral unit. Based on data collected using a telephone survey of 1,250 households in November 2007 and on data from the Central Statistics Office in the Republic of Ireland and the Northern Ireland Statistics and Research Agency.

The thematic choropleth maps are presented in Figures 1, 2, 3 and 4. The density of households with a pet dog, and with a pet cat, in Ireland, the Dublin area and the Belfast area are presented in Figures 1 and 2, respectively. The proportion of households with a pet dog, and with a pet cat, in Ireland, the Dublin area and the Belfast area are presented in Figures 3 and 4, respectively.

## Discussion

Spatial maps and analyses have previously been used to present disease spread in pet dogs [22] and cats [23]. Some studies have used human demographic figures to predict overall populations of pet dogs [1,10,24] and cats [1,24], but these results were not used to give an overall spatial description of the population. A study of cat ownership in Australia [25] displays a spatial representation of the proportion of cat owners, but only in the Sydney area and predictive factors were not considered. In our study, we used predictive probabilities of socio-demographic factors to determine the population of households that own a pet. These methods, as a means of demonstrating spatial distribution of pet ownership, have not been described previously.

One of the most dominant features of the maps in Figures 1 and 2 is the contrast between the densities of households owning dogs in urban areas with that of rural areas. The density of dog owning households is much higher in urban than rural districts. However, the spatial patterns are clearly different when considering the spatial distribution of the proportion of dog owning households (Figure 3), with this proportion being lower in urban compared to rural districts. These same features are also dominant in the cat owning households of Ireland (Figure 4). Cat owning households are sparse in rural areas (Figure 2), but the proportion of cat owning households is higher in urban districts (Figure 4). These findings can be explained somewhat by the difference in the density of households throughout Ireland. The number of households in inner-city electoral divisions (EDs) was as high as 10,581 per ED, whereas rural EDs were as low as 23 per ED [26].

In the large urban areas of Dublin and Belfast, there is an increase in the proportion of pet dog owning households in suburban districts compared to inner-city districts. In the UK, dog ownership has been shown to be associated with the presence of a garden [1], which may offer some explanation for the differences observed here. Suburban households generally have greater access to gardens and open green areas such as parks compared to inner city dwellings. These regional differences in pet dog ownership should be considered when setting up facilities for dog owners, and also when implementing animal control practices like dog warden numbers and disease surveillance.

In Ireland, most cats are adopted directly from the free-roaming population [21], which is likely to affect the spatial distribution of owned pet cats. The free-roaming population may be larger in suburban districts, given larger open spaces for cat colonies to form and greater access of free-roaming cats to houses through gardens. In inner-city apartment dwelling, access to households would be limited to ground floor apartments and there are fewer open spaces for colony formation.

This study was constrained by restricted access to census data as a consequence of data protection concerns [27]. Consequently, we were unable to predict pet ownership using more than one of the household factors that were previously identified as important [21]. Given this constraint, this study was conducted using household factors that were likely of greatest biological relevance to our study. Greater detail during mapping, and greater precision during estimation, would have been possible if all collected data had been available from the ROI and NI censuses. Also, projected estimates for NI household figures for 2006 were used instead of the actual census figures in 2001; as a consequence, there is less certainty about the 2006 NI (compared with ROI) estimates. The decision to use these figures was based on the aim to produce a uniform map reflecting the situation on the island of Ireland at a single point in time (namely, 2006). Spatial predictive probability modelling was of value in the current study, enabling us to utilise publicly available data, and to extrapolate results to both the surveyed and non-surveyed households.

## Conclusions

This study presents the spatial distribution of pet dogs and pet cats on the island of Ireland. It also provides an insight into the spatial relationship between human households and the density of pet dogs and pet cats. Knowledge of the spatial distribution of the baseline, normal population is important as it provides information when determining the incidence of disease and when comparing stray pet numbers to owned pets. With this type of objective data, it is possible to make informed decisions and recommendations when analysing disease prevalence and risk of disease spread through the population. For example, if an outbreak of rabies were to occur in the pet population in Ireland, this information could inform subsequent investigations with knowledge of the expected population size and distribution of the pet dog population. This information is also beneficial in instigating veterinary care and product marketing, based on objective information about the density of pet ownership in given areas.

## Methods

### 2.1 Data sources

#### 2.1.1 Pet-related data

This study was conducted using pet-related data collected previously [21]. Briefly, in this earlier study, a questionnaire was administered in 2007 to collect data about the demographics of households in Ireland and their dogs and cats. The questions related to location, building structure, social class, nationality and family structure of the household, and the sex, age and source of each pet dog and/or cat.

Restriction in the availability of detailed census data, due to data protection legislation [27], meant that we were unable to combine all of the household factors previously identified as important factors of pet ownership [21]. Therefore, these factors were screened to identify the one of greatest biological relevance, separately for pet dog and pet cat ownership. For pet dog ownership, 'household composition' was selected, noting that family structure is deemed influential for households when deciding whether to obtain a pet (Downes et al., unpublished). For pet cat ownership, 'house type' was selected, noting that cats tend to stray into a household [21]. Building type is likely to influence whether this occurs. In the current study, these two variables were categorised in a manner that matched those used by the organisations from which the human data was obtained (see 2.1.2). We developed two univariable logistic regression models, one each for the outcome variables pet dog ownership and pet cat ownership. In the pet dog ownership model, household composition was the independent variable. In the pet cat ownership model, the independent variable was house type. The outputs from these models were used to determine the predicted probabilities for pet dog and pet cat ownership in each of the categories of each independent variable. Statistical analyses were conducted using StataSE^® ^version 11 (StataCorp, College Station, TX, USA).

#### 2.1.2 Human data

Several data sources were used to obtain data on household composition and house type, including the Central Statistics Office [28,29] in the ROI, and the Northern Ireland Statistics and Research Agency (NISRA) [30-32] in NI:

a. *The 2006 census in Ireland*. In ROI, the latest nationwide census was conducted by the CSO on Sunday 23 April 2006. These data were used to produce the 2006 Small Area Population Statistics (SAPS), including house type [28] and household composition [29] by electoral division (ED; the smallest legally defined administrative area in the ROI).

b. *The 2001 census in Northern Ireland*. In the United Kingdom, of which NI is part, the latest nationwide census was conducted on Sunday 29 April 2001. In NI, the census is conducted by the NISRA. Data are available about house type [30] and household composition [31] by electoral ward. Electoral wards are the key building block of UK administrative geography, being the spatial units used to elect local government councillors in district council areas in Northern Ireland.

c. *Projected 2006 data for Northern Ireland*. Aggregated data (but not by ward) were available from the NISRA on the estimated number of households by household composition (but not house type) for 2006 [32].

#### 2.1.3 Map data

An ED map for ROI was obtained from UCD Urban Institute Ireland [33] and a ward map for NI from the NISRA [34].

### 2.2 Data management and analysis

The ED map for ROI was appended to the ward map for NI in ArcMap^® ^version 9.2 ERSI™, to create a single final electoral unit (EU) map for Ireland. Then, the census and map data were checked to ensure consistency with all ED and ward names. In ROI, some EDs in the census data had been aggregated; therefore, we also combined these EDs in the map data to create the final EU map.

In ROI, the predicted probabilities (from 2.1.1 above) for pet dog ownership by household composition and pet cat ownership by house type were applied to the 2006 census data (from 2.1.2a above) to estimate the total numbers, in 2006, of pet dog owning households per ED and pet cat owning households per ED. In NI, several steps were conducted. Firstly, the total numbers of pet dog owning and pet cat owning households per ward in 2001 was estimated based on the predicted probabilities (from 2.1.1) and the 2001 census data. Then, the percentage changes in aggregated measures (household composition) between 2001 and 2006 were used to provide estimates, per ward, of the number of households in 2006. The estimated number of pet dog and pet cat owning households per ward were then determined based on the estimated proportional change in the number of households per ward between 2001 and 2006. These ROI and NI data were combined, providing an estimate of numbers of households, pet dogs and pet cats by EU in 2006 (the 'population table'). All data cleaning and management were completed using Microsoft Excel 2007^® ^(Microsoft Corporation, Redmond, WA, USA).

The final EU map and 'population table' were combined using ArcMap^® ^version 9.2 ERSI™ to create a GIS database. Choropleth maps were created showing the proportion of households owning a pet dog or pet cat, and the density of pet dog or pet cat owning household in each ED. For mapping purposes, quintiles were selected as the most appropriate classification groups. Maps were created for Ireland, and for the two major cities, Dublin and Belfast.

## Authors' contributions

MD was involved in conceiving the study, coordinated the collection of the data. MD also carried out the statistical analysis, spatial map production, and drafted the manuscript. TAC participated in the analysis of the data determining the correct statistical tools to be used. DMC participated in the design and production of the final spatial maps. GMG participated in the acquisition of map data and design of the final maps. SJM was involved in conceiving the study, and participated in its design and coordination and helped to draft the manuscript. All authors read and approved the final manuscript.
